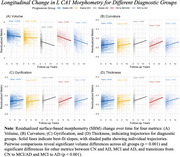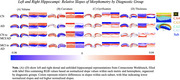# Longitudinal Surface‐Based Morphometry Changes in the Hippocampus in Dementia

**DOI:** 10.1002/alz70856_101579

**Published:** 2025-12-24

**Authors:** Salah Aziz, Romeo Penheiro, Cassandra Morrison, Peter Zhukovsky, John AE Anderson

**Affiliations:** ^1^ Carleton University, Ottawa, ON, Canada; ^2^ University of Houston, Houston, TX, USA; ^3^ Centre for Addiction and Mental Health, Kimel Family Translational Imaging Genetics Laboratory, Toronto, ON, Canada

## Abstract

**Background:**

The hippocampus plays a critical role in Alzheimer's disease (AD), marked by brain atrophy and cognitive decline. AD pathology begins during the transition from healthy aging to mild cognitive impairment (MCI) and eventually AD, with memory impairment as a hallmark. While hippocampal volume reductions are well‐documented, surface‐based morphometric (SBM) features—curvature, gyrification, and thickness—remain less understood.

**Method:**

T1‐weighted MRI data from 3.51 average annual scans (5,263 timepoints) across four phases of the Alzheimer's Disease Neuroimaging Initiative (ADNI) were analyzed using HippUnfold, a hippocampal subfield segmentation tool. Individuals (CN: 475; MCI: 673; AD: 269) were grouped by final clinical diagnosis to track cognitive trajectories: stable/non‐progressors (*n* = 1017) and progressors (*n* = 301). Linear mixed effects models adjusted for age, education, sex, scanner‐site, and eTIV evaluated hippocampal volume and surface metrics (CA1–CA4, DG, Subiculum, SRLM, and Cysts), with CN as the intercept.

**Results:**

Focusing on CA1, the region most vulnerable to early AD, the stable AD group showed significant volume reductions (β = ‐1.05, *p* < .001) compared to CN (β = 3.88, *p* < .001). All SBM metrics (curvature, gyrification, and thickness) showed significant changes, with curvature in CN (β = ‐0.48, *p* < .001) and AD (β = 0.01, *p* < .001). Time‐dependent interactions showed volume reductions in CN (β = ‐0.04, *p* < .001) and MCI (β = ‐0.07, *p* < .001), and increases in SBM metrics (all *p*'s < .001). Cognitive domain analyses showed volume changes primarily affect memory and executive functioning, while SBM metrics influence language and visuospatial ability. Curvature influenced language (61.76%, *p* < .001) and visuospatial ability (32.35%, *p* < .001), while thickness affected language (57.14%, *p* < .001) and visuospatial ability (35.71%, *p* < .001).

**Conclusion:**

Hippocampal volume reductions are well‐established markers of AD, but surface‐based features like curvature, gyrification, and thickness provide additional insights, revealing changes that volume alone may miss. These findings highlight the importance of integrating surface‐based metrics with volumetric analyses to improve understanding of disease mechanisms and interventions.